# Activity of fluconazole and its Cu(II) complex towards *Candida* species

**DOI:** 10.1007/s00044-014-1275-7

**Published:** 2014-10-09

**Authors:** Adam Ząbek, Justyna Nagaj, Agnieszka Grabowiecka, Ewa Dworniczek, Urszula Nawrot, Piotr Młynarz, Małgorzata Jeżowska-Bojczuk

**Affiliations:** 1 Department of Chemistry, Wroclaw University of Technology, Wybrzeże Wyspiańskiego 27, 50-370 Wrocław, Poland; 2 Faculty of Chemistry, University of Wrocław, Joliot-Curie 14, 50-383 Wrocław, Poland; 3 Department of Microbiology, Wrocław Medical University, Chałubińskiego 4, 50-386 Wrocław, Poland

**Keywords:** Fluconazole–Cu(II) complex, *Candida* spp., Azole resistance, MIC determination

## Abstract

*Candida* species, although they are present as commensal organisms in the digestive tract of healthy individuals, can produce a broad spectrum of serious illnesses in compromised hosts. Fluconazole, a water-soluble triazole with bioavailability greater than 90 %, has been extensively used to treat a wide range of *Candida* infections. However, a growing resistance of microorganisms in the treatment leads to the discovery of new drugs or modifications of existing ones. The aim of the present study was to investigate whether coordination of Cu(II) ions to fluconazole affects its antifungal activity. The in vitro susceptibility tests and antifungal studies were performed with two *Candida* spp.: *Candida* *glabrata* and *Candida albicans*. Overall, 34 strains of the former and 16 strains of the latter were treated with fluconazole, its Cu(II) complex and free Cu(II) ions. The obtained MIC values in 16 cases of the *C. glabrata* and in 5 cases of the *C. albicans* were lower for the complex in comparison to the drug. This implies that the complex is more effective against particular strains than the parent drug. The most significant improvement in the complex drug efficacy was observed for fluconazole-resistant species.

**Electronic supplementary material:**

The online version of this article (doi:10.1007/s00044-014-1275-7) contains supplementary material, which is available to authorized users.

## Introduction

In the last decades, a significant increase in incidence of opportunistic systemic fungal infections has been observed (Pfaller and Diekema, 2007). Although many of them could be successfully cured with available antifungal agents, the mortality due to systemic mycoses is still very high (30–90 %). The population of patients at risk is increasing and embraces mostly immunocompromised patients, particularly with HIV/AIDS, after bone marrow or solid organ transplantation, cancer patients undergoing chemotherapies, intensive care unit patients and preterm neonates (Sobel, 1992; Abi-Said *et al*., 1997; Ables *et al*., 2000; Alexander *et al*., 2005; Vazquez and Sobel, 2002; Tscherner *et al*., 2011; Kaufman, 2008).

Invasive mycoses can be caused by a broad spectrum of opportunistic fungal pathogens, the most important of which being members of the *Candida* genus. They represent the fourth most frequent pathogen isolated from the blood. The *Candida* represents a part of commensal flora of the gastrointestinal tract of 60–90 % of healthy human population. On the other hand, they are responsible for many types of superficial as well as deep seated infections, e.g. oral and vulvovaginal candidosis or candidaemia. *Candida albicans* is the species most frequently isolated from infection cases; however, the role of the “non-*albicans*” species, such as *Candida glabrata*, *Candida parapsilosis*, *Candida tropicalis* and *Candida krusei*, is growing systematically (Biswas *et al*., 2007; Eggimann *et al*., 2003; Jarvis, 1995; Silva *et al*., 2012). An important characteristic of many “non-*albicans*” species is their low susceptibility or even resistance to the frequently used antimycotics. Infections caused by them may result from the repeated and prolonged exposure to the same antifungal drugs which lead to the selection of resistant strains.

Fluconazole (FLZ, 2-(2,4-difluorophenyl)-1,3-*bis*(1H-1,2,4-triazol-1-yl)propan-2-ol) is one of the most widespreadly used antifungal agents (Charlier *et al*., 2006; Dery and Hasbun, 2011). Owing to its excellent pharmacokinetics, spectrum of activity, bioavailability, low toxicity and lack of interaction with other drugs, fluconazole has so far been used to treat more than 100 million people in the world (Charlier *et al*., 2006; Dery and Hasbun, 2011; Sabatelli *et al*., 2006; Löffler *et al*., 1997). Since 1990, when fluconazole was for the first time introduced to therapy, a variety of resistance mechanisms, including overexpression of various genes, has been observed (Charlier *et al*., 2006; Löffler *et al*., 1997; Franz *et al*., 1998; Parkinson *et al*., 1995; Orozco *et al*., 1998).

**Fig. 1:**
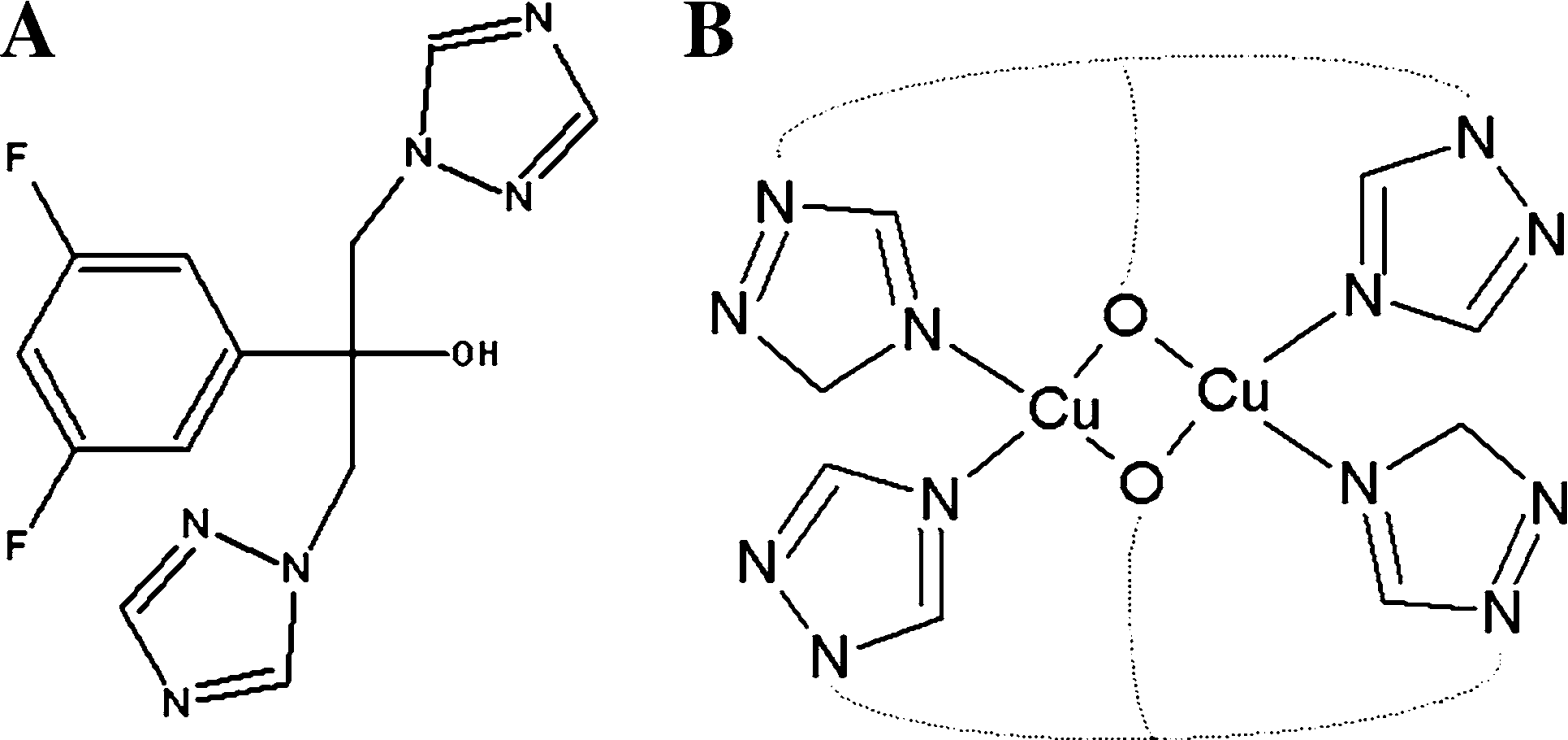
**a** The structure of fluconazole molecule **b** The schematic model of coordination patterns proposed for the FLZ-Cu system in water solution around pH 7.0 (conditions for microbiological experiments)

A growing interest in metal ion complexes as antimicrobial, diagnostic or chemotherapeutic agents has been observed for many years. Therefore, the aim of this paper was to test the antifungal properties of a Cu(II) ion complex with fluconazole (Fig. 1a), especially towards the drug-resistant *Candida* species. Under physiological conditions, a binuclear complex is formed, in which two Cu(II) ions are linked via two fluconazole molecules, engaging in the coordination process its nitrogen atoms as well as bridging oxygen atoms, which additionally stabilizes the complex structure. The structure of the [Cu_2_(fluconazole)_2_(H_2_O)_2_]^2+^ complex (FLZ-Cu), which exists both in solution and in the solid state, was described in detail in our previous paper (Nagaj *et al*., 2012). The proposed scheme of the coordination mode of the complex species in solution at the pH of around 7.0 (conditions for microbiological experiments) is presented in Fig. 1b.

## Materials and methods

### Clinical isolates

Fifty clinical isolates of *Candida* (16 strains of *C. albicans* and 34 strains of *C. glabrata*) were isolated from stool, urine, blood, wound, catheters, sputum and throat swabs. They were identified by ID32 (bioMerieux) test in the Department of Microbiology of the Wrocław Medical University. All strains were preserved in −80 °C in Tryptic Soy broth (Sigma-Aldrich), supplemented with 10 % glycerol (Sigma-Aldrich) and subcultured onto Sabouraud’s dextrose agar (Sigma-Aldrich) for 24 h to ensure viability and purity prior testing.

### Assay media and solutions

For antifungal susceptibility testing, the modified RPMI 1640 medium without bicarbonate (Sigma-Aldrich) buffered to pH 7.0 with 0.165 M 3-(*N*-morpholino)-propanesulfonic acid (Sigma-Aldrich) and supplemented with glucose to final concentration of 2 % per litre (RPMI 1640 2 % G) was used.

All used solutions were prepared according to Good Manufacturing Practice. Fluconazole (Sigma-Aldrich) was dissolved in double-strength culture medium RPMI 1640 2 % G at concentration of 256 μg/mL. The blank solution of free copper ions was prepared in the same way. Standard solution of copper ions complex with fluconazole was prepared by adding Cu(II) chloride (Sigma-Aldrich) to solution of fluconazole in 1:1 molar ratio.

### Susceptibility testing

Susceptibility testing of each isolate was performed according to the EUCAST (European Committee on Antimicrobial Susceptibility Testing) broth microdilution method (Rodriguez-Tudela *et al*. 2002), to establish minimum inhibitory concentrations (MICs) of antifungal agents. Sterile 96-well flat-bottom plates containing 100 μL of the twofold serial dilutions of FLZ, FLZ-Cu or Cu(II) ions in double-strength RPMI 1640 medium 2 % G (Sigma-Aldrich) were inoculated with 100 μL of yeast suspensions containing 1–5 × 10^5^ cfu/mL. Drug and complex dilutions were ranged from 0.125 to 128 μg/mL. The plates were incubated at 37 °C for 24 h. The fungal growth was measured at wavelength 530 nm by TECAN Microplate Reader Sunrise^TM^. All assays were performed at least six times, apart from four strains of *C. glabrata* (1941, 1973, 2098, 2221) and two strains of *C. albicans* (2210, 2211), which exhibited the best growth reduction in the presence of FLZ-Cu. For these strains, assays were conducted twelve times, and the results were confirmed by *t* test for significance (*p* < 0.05), using STATISTICA 10.0 for Windows (StatSoft, Poland). The data are expressed as mean values, and they are the average of 6 or 12 independent experiments, done in triplicate.

The strains were classified according to the clinical breakpoints (CBPs) developed by EUCAST (Version 6.1, valid from 2013 to 03-11, www.eucast.org/) as susceptible (S) [MIC ≤ 2 mg/L for *C. albicans* and MIC ≤ 0.002 mg/L for *C. glabrata*], resistant (R) [MIC ≥ 4 mg/L for *C. albicans* and MIC ≥ 32 mg/L for *C. glabrata*] and intermediate susceptible (I) [2 mg/L < MIC < 4 mg/L for *C. albicans* and 0.002 mg/L < MIC < 32 mg/L for *C. glabrata*] (Espinel-Ingroff *et al*., 2013).

## Results

### Susceptibility of strains and species-specific clinical breakpoints

Antifungal susceptibility tests were performed on fifty yeast strains of *Candida* spp. For each strain and investigated agent, the percentage distribution of growth reduction and the MICs values were determined. The obtained in vitro results revealed different susceptibility for both *C. glabrata* and *C. albicans* strains. According to CBPs for fluconazole, thirteen *C. albicans* strains were classified as susceptible, three *C. albicans* and four *C. glabrata* were resistant, while thirty *C. glabrata* strains were intermediate susceptible (Supplementary materials, Table S1).

### The antifungal effect of the fluconazole–Cu(II) complex

**Fig. 2:**
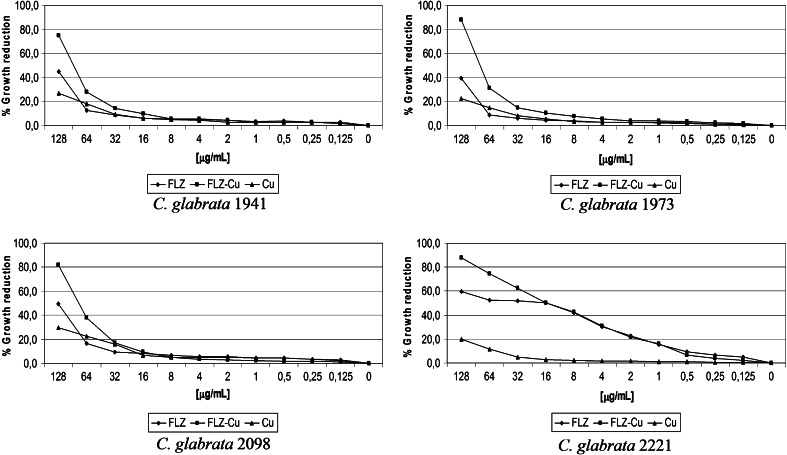
Distribution of percentage growth reduction for FLZ and FLZ-Cu for 4 strains of *C. glabrata* (1941, 1973, 2098, 2221)

For the purpose of establishing the antifungal effect of the FLZ-Cu complex, all tested strains were classified to an appropriate susceptibility group. Table S1 presents the percentage distribution of growth reduction, and the MICs values obtained for examined strains treated with FLZ and FLZ-Cu. The MIC values indicated that in 16 cases of *C. glabrata* strains, the studied complex only twice reduced those values. A modest effect was achieved for three out of four resistant (R) *C. glabrata* strains (1941, 1973, 2098). The MIC values obtained for them were for FLZ above 128 μg/mL (Fig. 2), whereas the susceptibility test result for FLZ-Cu was exactly equal to 188.22 μg/mL. Moreover, the complex revealed a slightly higher influence (*p* < 0.05) on the percentage growth reduction than FLZ in the range of concentrations 4–128 μg/mL, 16–128 μg/mL and 32–128 μg/mL for *C. glabrata* 1973, 1941 and 2098, respectively (Table S1, highlighted orange background). This effect was also observed for other strains (e.g. *C. glabrata* 2221), which exhibited the same MIC values for FLZ and for its cupric complex (Fig. 2; *p* < 0.05 for range of concentration 32-128 μg/mL).

**Fig. 3:**
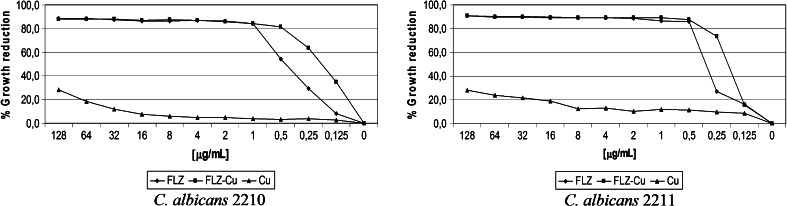
Distribution of percentage growth reduction for FLZ and FLZ-Cu complex for 2 strains of *C. albicans* (2210, 2211)

Only for five tested strains of *C. albicans*, which were susceptible, the complex exhibited a little better antifungal activity than for a free ligand. The percentage growth reduction distribution for two of them is presented in Fig. 3 (Table S1, highlighted orange background). As it can be seen, similarly to the instance of *C. glabrata*, at a low concentration (0.125-0.5 μg/mL FLZ-Cu), the complex was about 10–40 % more effective than FLZ (*p* < 0.05). This effect was not observed among the drug-resistant strains of *C. albicans* (Table S1). Furthermore, for *C. albicans* 2218, the activity of FLZ-Cu was much lower than that of uncomplexed drug.

### The antifungal effect of Cu(II) ions

In the case of copper ions studies, the obtained results showed the fungal growth reduction on the level of 10–25 % for 30 strains, 26–30 % for 15 strains and >35 % for 5 strains. For those strains of *C. glabrata* which revealed good susceptibility to the FLZ-Cu complex (1941, 1973, 2098, 2221), the values of growth reduction for copper ions were less significant, ranging from 20 to 30 % (Fig. 2). A similar effect was obtained for strains of *C. albicans* (2210, 2211), for which those values reached 28.5 % (Fig. 3, Table S1).

## Discussion

Copper complexes have gained a growing interest as pharmaceuticals to be used as diagnostic, antimicrobial, antiviral, anti-inflammatory or antitumor agents (Gielen and Tiekink, 2005; Zhang and Lippard, 2003; Ming, 2003; Iakovidis *et al*., 2011; Weder *et al*., 2002; Regtop and Biffin, 1994; Tisato *et al*., 2010). There are a number of antibiotics named “metalloantibiotics” that require metal ions to act properly, such as bleomycin, streptonigrin, bacitracin and albomycin. The coordinated metal ions play an important role in maintaining a suitable structure and function of these antibiotics (Ming, 2003). It has been shown that copper complexes with non-steroidal anti-inflammatory drugs reveal enhanced anti-inflammatory and antiulcerogenic activity, as well as reduced gastrointestinal toxicity in comparison with uncomplexed drugs (Iakovidis *et al*., 2011; Weder *et al*., 2002; Regtop and Biffin, 1994). The best example is the cupric complex of indomethacine, which exhibits a higher anti-inflammatory activity and lower toxicity than the initial agent, i.e. in a metal-free form (Regtop and Biffin, 1994). Other studies presented in literature have focused on chemotherapeutic effects of copper complexes and their use in antitumor therapy, where one of the best examples is bleomycin (Ming, 2003; Tisato *et al*., 2010).

Taking into account the therapeutic potential of bioinorganic drugs, complexation of the known drugs to metal ions seems to also be an appropriate strategy for the design of antifungal agents. So far, the examples reported in the literature include the enhanced antifungal activity of the fluconazole-Ag(I) complex against *Saccharomyces cerevisiae*, *Mucor mucedo*, *Rhizopus tolonifer*, *Penicillium uniculosum and Aspergillus niger* in comparison with the metal-free form (Zhang *et al*., 2007). Furthermore, there are reports describing higher antifungal activity of other metal ions complexes (Shreaz *et al*., 2010; Ali *et al*., 2012). One of them is the paper concerning a good activity of copper and nickel complexes towards different strains of *Candida* (Ali *et al*., 2012).

The purpose of the presented research was to test the antifungal properties of the FLZ-Cu complex against the fungal strains of *C. glabrata* and *C. albicans*. It should be noted that during conducted study in RMPI medium, the fraction of FLZ-Cu complex has decayed. The NMR studies (data not shown) revealed that copper ions interact with FLZ, and the small remaining fraction of FLZ-Cu is still able to show better activity than FLZ alone. This is clearly seen in Figs. 2 and 3, where the copper ions complexed to the ingredients of the medium exhibit lower activity than those bound to fluconazole. Thus, the effect of FLZ-Cu in the absence of medium components should be much greater.

The obtained results allow to assume that FLZ-Cu complex could be used for exterior purposes as a component of ointment. In this case, the daily doses of the FLZ (the number of repetitions of lubrication), particularly in case of invasive fungal infections, could be significantly reduced by applying its complexed form.

The molecular mechanism of fluconazole action is well known (Charlier *et al*., 2006). It involves the reduction of ergosterol production of one of the major components of yeast cell membrane, by blocking the activity of the P450 enzyme. A major problem in the treatment of fungal infections is the constantly increasing number of strains resistant to the drugs used. For FLZ, there are three known mechanisms of resistance (Charlier *et al*., 2006; Löffler *et al*., 1997; Franz *et al*., 1998; Parkinson *et al*., 1995; Orozco *et al*., 1998). One of them is associated with the overactivity of the efflux pump, which significantly reduces the intracellular concentration of the drug below the effective level. This overactivity is related to the overexpression of the two gene families (CDR and MDR), which can contribute to an increase of the MIC values (Parkinson *et al*., 1995). As reported by Sanguinetti *et al*. (2005), among the strains of *C. glabrata,* the MIC values greater than 32 μg/mL are strongly correlated with the upregulation of efflux transporters. Although with no doubt, more detailed studies are required to explain the action of the FLZ-Cu complex; we can suppose that a complexed form of the drug is able to deliver more molecules of fluconazole into the cell interior, thus overcoming the overactivity of efflux pumps. The process of formation of FLZ-Cu complexes considerably reduces the polarity of the metal ion because of the partial sharing of its positive charge with the ligand donor groups. Such chelation could increase the lipophilic character of the central metal ion. This can be helpful for fluconazole complex in the penetration through the lipid layer of the cell membrane (Gölcü and Dolaz, 2006). On the other hand, the observed effects could not be enough significant to warrant that FLZ-Cu is able to deliver more drug into the cell interior and thus overcome the activity of efflux pumps.

At the same time, the mechanism associated with ROS generation can be disregarded as it has been reported earlier (Nagaj *et al*., 2012). An additional proof of absence of ROS activity in all isolates is small influence of free copper ions on their growth reduction. Copper concentrations used in those studies were not toxic for growth of the investigated yeast of genus *Candida* spp. According to literature reference data, the toxic concentration of free copper ions is over 1.5 g/L (Adamo *et al*., 2012; Avery *et al*., 1996). Therefore, the probable mechanism of impact of Cu(II) ions on the investigated strains should have another reason.

## Conclusion

An increase a number drug-resistant strains of the *Candida* species caused systemic invasive fungal infections has become the inspiration to perform studies of antifungal activity of fluconazole modified by copper ions binding. The antifungal activity of FLZ-Cu was elaborated and explored. However, only slightly improved effect on the drug-resistant strains of *C. glabrata* and a moderate on susceptible *C. albicans* was observed. Despite the same value of MICs, the percentage growth reduction of individual strains of *C. glabrata* and *C. albicans* (free living planktonic forms of *Candida* spp.) was greater by approximately 10–40 % for the complex in comparison with the copper-free drug. The obtained results indicate modest activity of the FLZ-Cu complex for chosen strains but may help to define a new direction for studies of antifungal drugs.

## Electronic supplementary material

Below is the link to the electronic supplementary material.

Growth reduction rate and minimum inhibitory concentration (MIC) values obtained for fluconazole and fluconazole–Cu(II) complex (1:1) for 50 isolates of *C. glabrata* and *C. albicans* by the EUCAST method. (DOC 267 kb)
