# Supplementary material for: Activity of fluconazole and its Cu(II) complex towards Candida species
Source: Med Chem Res. 2014 Oct 9;24(5):2005–10. doi: 10.1007/s00044-014-1275-7 (PMC4432085; doi:10.1007/s00044-014-1275-7)
Supplement: Supplementary file 1 — Growth reduction rate and minimum inhibitory concentration (MIC) values obtained for fluconazole and fluconazole–Cu(II) complex (1:1) for 50 isolates of C. glabrata and C. albicans by the EUCAST method. (DOC 267 kb) [file 44_2014_1275_MOESM1_ESM.doc]

**Table S1**

Growth reduction rate and minimum inhibitory concentration (MIC) values obtained for fluconazole fluconazole-Cu(II) complex and for copper ions for 50 isolates of *C. glabrata* and *C. albicans* by the EUCAST method.

| Species  Antifungal agent | | Concentration of antifungal agent (μg/mL) | | | | | | | | | | | MIC (μg/mL) | Susceptibility categories |
| --- | --- | --- | --- | --- | --- | --- | --- | --- | --- | --- | --- | --- | --- | --- |
|  |  | 128^*^ | 64 | 32 | 16 | 8 | 4 | 2 | 1 | 0.5 | 0.25 | 0.125 |  |  |
|  |  | 188.22** | 94.11 | 47.055 | 23.527 | 11.764 | 5.882 | 2.941 | 1.47 | 0.735 | 0.368 | 0.184 |  |  |
|  |  | 56.19*** | 28.095 | 14.047 | 7.024 | 3.512 | 1.756 | 0.878 | 0.439 | 0.219 | 0.110 | 0.055 |  |  |
| *C. glabrata* 1672 | FLZ | 93.8 | 88.6 | 79.1 | 68.9 | 34.3 | 22.1 | 15.6 | 7.9 | 7.1 | 5.5 | 2.1 | **16** | **I** |
|  | FLZ-Cu | 83.2 | 80.7 | 73.8 | 67.2 | 54.3 | 27.9 | 14.5 | 7.4 | 4.3 | 3.5 | 2.4 | **8** |  |
|  | Cu(II) | 29.7 | 18.3 | 13.9 | 10.6 | 9.1 | 6.9 | 6.2 | 6.1 | 4.5 | 4.6 | 2.9 | **-** |  |
| *C. glabrata* 1702 | FLZ | 69.8 | 61.8 | 59.2 | 49.6 | 35.3 | 18.3 | 8.3 | 6.3 | 5.5 | 4.1 | 3.5 | **32** | **I** |
|  | FLZ-Cu | 78.6 | 75.4 | 69.9 | 63.4 | 48.9 | 27.7 | 11.3 | 5.9 | 5.2 | 3.4 | 3.2 | **16** |  |
|  | Cu(II) | 34.6 | 28.6 | 17.3 | 9.3 | 6.6 | 5.7 | 4.1 | 3.4 | 3.4 | 3.1 | 0.6 | **-** |  |
| *C. glabrata* 1718 | FLZ | 89.0 | 88.1 | 83.3 | 75.5 | 30.3 | 16.3 | 9.1 | 7.6 | 5.2 | 4.1 | 2.6 | **16** | **I** |
|  | FLZ-Cu | 84.5 | 82.4 | 83.6 | 79.6 | 49.0 | 22.0 | 13.7 | 6.9 | 5.8 | 5.0 | 2.6 | **16** |  |
|  | Cu(II) | 40.5 | 18.5 | 12.3 | 7.0 | 4.9 | 4.7 | 3.7 | 2.4 | 1.4 | 1.7 | 1.6 | **-** |  |
| *C. glabrata* 1728 | FLZ | 89.6 | 88.3 | 80.7 | 64.8 | 36.4 | 26.8 | 14.2 | 7.2 | 7.8 | 5.6 | 4.3 | **16** | **I** |
|  | FLZ-Cu | 95.9 | 88.1 | 80.8 | 74.1 | 51.4 | 32.7 | 21.3 | 9.0 | 6.4 | 4.9 | 3.7 | **8** |  |
|  | Cu(II) | 29.9 | 23.2 | 10.6 | 4.2 | 3.1 | 2.4 | 1.7 | 1.0 | 1.0 | 0.9 | 0.6 | **-** |  |
| *C. glabrata* 1739 | FLZ | 90.1 | 89.8 | 88.8 | 81.0 | 53.3 | 21.6 | 8.3 | 4.3 | 3.6 | 3.1 | 2.2 | **8** | **I** |
|  | FLZ-Cu | 87.6 | 87.8 | 85.6 | 84.5 | 74.4 | 38.1 | 16.1 | 5.5 | 5.4 | 4.7 | 4.6 | **8** |  |
|  | Cu(II) | 33.0 | 24.0 | 17.4 | 8.6 | 5.1 | 4.0 | 3.7 | 1.3 | 1.1 | 1.0 | 0.7 | **-** |  |
| *C. glabrata* 1785 | FLZ | 83.8 | 81.9 | 81.8 | 59.3 | 25.4 | 14.0 | 10.8 | 7.9 | 5.6 | 3.7 | 2.3 | **16** | **I** |
|  | FLZ-Cu | 85.0 | 85.0 | 82.8 | 74.6 | 43.0 | 23.1 | 14.0 | 9.0 | 5.7 | 4.3 | 4.2 | **16** |  |
|  | Cu(II) | 34.9 | 24.9 | 15.4 | 7.5 | 5.1 | 5.6 | 2.8 | 2.2 | 1.9 | 1.4 | 0.3 | **-** |  |
| *C. glabrata* 1915 | FLZ | 93.2 | 92.9 | 92.7 | 92.4 | 88.8 | 67.9 | 41.4 | 29.3 | 13.4 | 7.3 | 4.7 | **4** | **I** |
|  | FLZ-Cu | 87.1 | 86.8 | 85.8 | 85.6 | 84.6 | 74.5 | 46.4 | 28.4 | 15.9 | 9.4 | 4.9 | **4** |  |
|  | Cu(II) | 43.9 | 33.1 | 27.9 | 14.1 | 7.3 | 5.6 | 4.2 | 3.9 | 3.5 | 2.5 | 1.5 | **-** |  |
| *C. glabrata* 1941 | FLZ | 44.8 | 12.7 | 8.9 | 6.3 | 5.2 | 5.1 | 4.6 | 3.3 | 3.6 | 2.6 | 2.5 | **> 128** | **R** |
|  | FLZ-Cu | 74.7 | 27.6 | 14.4 | 9.9 | 5.2 | 5.3 | 4.3 | 2.6 | 2.7 | 2.7 | 1.4 | **128** |  |
|  | Cu(II) | 26.9 | 18.3 | 9.4 | 5.9 | 5.0 | 4.2 | 2.7 | 2.6 | 2.5 | 2.5 | 1.9 | **-** |  |
| *C. glabrata* 1959 | FLZ | 89.8 | 86.9 | 85.0 | 69.6 | 32.5 | 18.6 | 7. 5 | 5.0 | 3.1 | 1.8 | 0.2 | **16** | **I** |
|  | FLZ-Cu | 80.9 | 78.3 | 74.6 | 70.0 | 47.7 | 28.3 | 12.0 | 6.2 | 5.9 | 4.4 | 3.3 | **16** |  |
|  | Cu(II) | 31.7 | 18.8 | 12.1 | 3.6 | 1.7 | 2.0 | 1.2 | 1.4 | 2.1 | 1.6 | 0.4 | **-** |  |
| *C. glabrata* 1973 | FLZ | 39.1 | 8.9 | 6.0 | 4.5 | 3.8 | 3.0 | 2.9 | 2.0 | 1.8 | 1.1 | 1.1 | **> 128** | **R** |
|  | FLZ-Cu | 87.8 | 31.3 | 14.8 | 10.5 | 7.4 | 5.6 | 3.8 | 3.6 | 3.4 | 2.2 | 1.4 | **128** |  |
|  | Cu(II) | 22.1 | 14.9 | 8.1 | 5.3 | 3.5 | 2.9 | 2.6 | 2.7 | 2.0 | 1.3 | 0.4 | **-** |  |
| *C. glabrata* 2011 | FLZ | 89.8 | 88.5 | 87.7 | 87.6 | 84.1 | 34.1 | 12.6 | 6.7 | 5.0 | 4.0 | 3.8 | **8** | **I** |
|  | FLZ-Cu | 75.9 | 65.5 | 57.5 | 49.8 | 39.9 | 32.9 | 18.5 | 9.8 | 3.6 | 2.4 | 2.6 | **16** |  |
|  | Cu(II) | 33.1 | 27.3 | 20.8 | 10.1 | 5.2 | 2.1 | 1.8 | 1.3 | 1.2 | 0.7 | 0.5 | **-** |  |
| *C. glabrata* 2016 | FLZ | 76.9 | 75.2 | 74.8 | 67.9 | 45.8 | 30.5 | 17.9 | 7.6 | 5.2 | 4.9 | 2.6 | **16** | **I** |
|  | FLZ-Cu | 79.4 | 75.3 | 71.3 | 69.4 | 51.8 | 33.2 | 18.1 | 5.8 | 3.9 | 3.0 | 2.2 | **8** |  |
|  | Cu(II) | 29.2 | 17.4 | 10.5 | 4.0 | 3.2 | 2.6 | 1.9 | 1.7 | 1.4 | 1.4 | 0.3 | **-** |  |
| *C. glabrata* 2019 | FLZ | 91.7 | 90.8 | 89.5 | 76.7 | 42.0 | 30.8 | 23.7 | 16.2 | 14.8 | 9.9 | 6.1 | **16** | **I** |
|  | FLZ-Cu | 90.8 | 88.4 | 81.0 | 80.3 | 50.1 | 33.8 | 28.0 | 19.9 | 17.2 | 12.7 | 8.3 | **8** |  |
|  | Cu(II) | 11.6 | 8.1 | 5.5 | 2.9 | 2.7 | 2.5 | 2.3 | 1.8 | 1.5 | 1.5 | 1.3 | **-** |  |
| *C. glabrata* 2087 | FLZ | 86.7 | 85.1 | 77.0 | 42.9 | 26.4 | 20.8 | 11.2 | 4.9 | 4.0 | 2.9 | 2.1 | **32** | **I** |
|  | FLZ-Cu | 78.1 | 73.2 | 73.3 | 62.7 | 39.7 | 17.9 | 9.6 | 2.6 | 2.9 | 1.3 | 1.8 | **16** |  |
|  | Cu(II) | 28.3 | 27.6 | 26.2 | 28.5 | 25.0 | 11.9 | 8.9 | 8.9 | 5.8 | 4.4 | 1.9 | **-** |  |
| *C. glabrata* 2098 | FLZ | 49.6 | 16.4 | 9.4 | 8.3 | 6.6 | 5.3 | 5.5 | 4.6 | 4.1 | 3.3 | 2.8 | **> 128** | **R** |
|  | FLZ-Cu | 82.1 | 38.0 | 16.8 | 9.2 | 5.0 | 3.5 | 2.7 | 2.2 | 1.9 | 1.4 | 1.3 | **128** |  |
|  | Cu(II) | 29.5 | 22.4 | 16.0 | 6.4 | 5.2 | 4.7 | 4.9 | 4.6 | 4.5 | 3.4 | 2.3 | **-** |  |
| *C. glabrata* 2103 | FLZ | 88.8 | 69.3 | 61.7 | 60.8 | 58.3 | 48.9 | 27.6 | 12.7 | 6.7 | 4.9 | 3.3 | **8** | **I** |
|  | FLZ-Cu | 88.9 | 86.2 | 79.0 | 74.9 | 65.6 | 52.8 | 27.0 | 11.0 | 6.7 | 4.2 | 3.7 | **4** |  |
|  | Cu(II) | 27.8 | 17.9 | 8.7 | 4.8 | 3.5 | 3.6 | 3.7 | 2.9 | 2.5 | 2.8 | 1.0 | **-** |  |
| *C. glabrata* 2170 | FLZ | 91.2 | 91.1 | 90.5 | 89.7 | 86.2 | 75.5 | 32.0 | 18.1 | 12.6 | 5.1 | 1.8 | **4** | **I** |
|  | FLZ-Cu | 93.7 | 89.4 | 85.8 | 81.9 | 77.0 | 66.3 | 39.3 | 21.6 | 15.2 | 11.8 | 8.6 | **4** |  |
|  | Cu(II) | 21.1 | 14.7 | 8.6 | 4.2 | 2.8 | 1.8 | 1.7 | 1.0 | 0.9 | 0.8 | 0.6 | **-** |  |
| *C. glabrata* 2192 | FLZ | 60.2 | 59.6 | 59.3 | 59.1 | 58.7 | 58.6 | 58.5 | 56.3 | 55.6 | 51.0 | 39.5 | **0.25** | **I** |
|  | FLZ-Cu | 86.4 | 82.3 | 73.5 | 66.2 | 64.2 | 61.3 | 60.0 | 58.2 | 58.1 | 55.8 | 45.5 | **0.25** |  |
|  | Cu(II) | 41.0 | 29.4 | 26.6 | 26.5 | 25.4 | 25.3 | 24.8 | 24.3 | 21.9 | 18.9 | 14.7 | **-** |  |
| *C. glabrata* 2205 | FLZ | 75.3 | 73.9 | 73.8 | 72.7 | 49.6 | 29.3 | 5.3 | 5.3 | 3.7 | 3.4 | 1.0 | **16** | **I** |
|  | FLZ-Cu | 81.5 | 80.8 | 80.4 | 78.3 | 72.7 | 30.6 | 29.8 | 24.9 | 24.1 | 23.6 | 19.3 | **8** |  |
|  | Cu(II) | 31.7 | 18.8 | 12.1 | 3.6 | 1.7 | 2.0 | 1.2 | 1.4 | 2.1 | 1.6 | 0.4 | **-** |  |
| *C. glabrata* 2206 | FLZ | 86.8 | 83.6 | 83.2 | 83.0 | 81.9 | 76.5 | 48.5 | 22.2 | 13.4 | 10.5 | 7.5 | **4** | **I** |
|  | FLZ-Cu | 93.6 | 88.7 | 87.6 | 85.9 | 84.2 | 79.9 | 54.6 | 19.6 | 10.2 | 9.2 | 7.8 | **2** |  |
|  | Cu(II) | 32.0 | 20.8 | 7.4 | 2.1 | 4.3 | 2.8 | 3.0 | 2.4 | 1.7 | 0.7 | 0.1 | **-** |  |
| *C. glabrata* 2207 | FLZ | 91.7 | 91.6 | 90.7 | 70.6 | 59.2 | 43.1 | 29.8 | 25.5 | 16.4 | 12.9 | 1.9 | **8** | **I** |
|  | FLZ-Cu | 89.0 | 88.5 | 87.8 | 83.9 | 66.9 | 47.4 | 26.1 | 25.0 | 23.3 | 10.9 | 5.8 | **8** |  |
|  | Cu(II) | 31.3 | 20.6 | 8.6 | 6.0 | 4.9 | 4.6 | 4.0 | 3.7 | 1.6 | 1.5 | 0.7 | **-** |  |
| *C. glabrata* 2220 | FLZ | 67.2 | 64.2 | 62.3 | 61.0 | 53.7 | 38.9 | 24.9 | 14.7 | 10.1 | 8.0 | 6.0 | **8** | **I** |
|  | FLZ-Cu | 90.0 | 57.6 | 51.5 | 50.2 | 49.8 | 37.5 | 23.3 | 11.6 | 7.2 | 5.9 | 4.0 | **16** |  |
|  | Cu(II) | 36.2 | 26.2 | 14.5 | 7.3 | 3.6 | 3.3 | 1.8 | 1.9 | 2.1 | 1.9 | 1.9 | **-** |  |
| *C. glabrata* 2221 | FLZ | 59.5 | 52.5 | 52.1 | 50.0 | 41.7 | 30.4 | 22.7 | 15.4 | 9.6 | 6.7 | 4.9 | **16** | **I** |
|  | FLZ-Cu | 87.8 | 74.3 | 62.7 | 50.3 | 42.3 | 30.8 | 21.6 | 15.8 | 6.6 | 3.9 | 2.3 | **16** |  |
|  | Cu(II) | 20.0 | 11.6 | 4.9 | 2.5 | 2.4 | 1.8 | 1.6 | 1.2 | 1.0 | 0.7 | 0.5 | **-** |  |
| *C. glabrata* 2222 | FLZ | 68.6 | 60.6 | 58.9 | 52.1 | 36.5 | 22.8 | 13.2 | 8.2 | 7.4 | 5.8 | 1.5 | **16** | **I** |
|  | FLZ-Cu | 80.3 | 67.8 | 60.4 | 53.9 | 41.5 | 28.3 | 10.5 | 3.0 | 2.9 | 2.4 | 1.5 | **16** |  |
|  | Cu(II) | 22.4 | 14.6 | 7.9 | 3.9 | 4.7 | 4.1 | 4.0 | 1.4 | 1.4 | 2.5 | 0.7 | **-** |  |
| *C. glabrata* 2223 | FLZ | 82.3 | 81.4 | 81.1 | 78.6 | 67.6 | 54.3 | 48.5 | 43.3 | 42.8 | 38.4 | 34.1 | **4** | **I** |
|  | FLZ-Cu | 86.1 | 86.5 | 85.2 | 80.2 | 71.8 | 52.9 | 33.3 | 32.6 | 32.2 | 30.9 | 27.1 | **4** |  |
|  | Cu(II) | 50.1 | 48.5 | 45.9 | 39.9 | 41.0 | 38.4 | 34.1 | 34.1 | 26.7 | 25.1 | 25.1 | **-** |  |
| *C. glabrata* 2224 | FLZ | 90.2 | 88.9 | 88.0 | 87.0 | 80.6 | 59.2 | 41.2 | 31.4 | 25.7 | 24.5 | 23.0 | **4** | **I** |
|  | FLZ-Cu | 90.3 | 87.6 | 86.6 | 85.3 | 81.2 | 57.3 | 39.2 | 31.1 | 13.3 | 8.8 | 8.3 | **4** |  |
|  | Cu(II) | 33.9 | 21.5 | 21.8 | 16.8 | 16.1 | 13.3 | 12.0 | 11.9 | 7.7 | 6.6 | 6.5 | **-** |  |
| *C. glabrata* 2226 | FLZ | 86.8 | 86.7 | 86.3 | 82.9 | 81.9 | 76.5 | 66.3 | 3.7 | 3.2 | 2.9 | 2.1 | **2** | **I** |
|  | FLZ-Cu | 91.6 | 90.9 | 89.7 | 88.4 | 85.3 | 82.6 | 56.3 | 9.7 | 3.1 | 1.9 | 1.8 | **2** |  |
|  | Cu(II) | 10.0 | 6.0 | 3.9 | 3.6 | 3.0 | 2.9 | 1.7 | 1.1 | 0.9 | 0.7 | 0.3 | **-** |  |
| *C. glabrata* 2228 | FLZ | 88.0 | 87.0 | 85.6 | 83.6 | 65.8 | 25.0 | 16.3 | 8.9 | 5.8 | 3.6 | 2.6 | **8** | **I** |
|  | FLZ-Cu | 88.8 | 88. | 86.7 | 84.3 | 67.5 | 31.5 | 20.6 | 13.1 | 7.3 | 4.7 | 3.4 | **8** |  |
|  | Cu(II) | 15.5 | 8.9 | 4.4 | 2.5 | 2.4 | 2.3 | 1.4 | 1.1 | 0.3 | 0.2 | 0.1 | **-** |  |
| *C. glabrata* 2229 | FLZ | 93.8 | 94.6 | 94.8 | 94.7 | 94.9 | 94.9 | 94.5 | 94.1 | 93.0 | 65.0 | 4.2 | **0.25** | **I** |
|  | FLZ-Cu | 93.9 | 94.5 | 95.1 | 94.9 | 95.3 | 95.4 | 95.2 | 94.0 | 92.8 | 44.3 | 6.3 | **0.5** |  |
|  | Cu(II) | 21.4 | 7.7 | 6.0 | 3.8 | 3.2 | 3.7 | 3.0 | 0.4 | 0.1 | 0.8 | 0.6 | **-** |  |
| *C. glabrata* 2230 | FLZ | 89.3 | 88.4 | 78.3 | 51.7 | 30.1 | 18.8 | 9.2 | 5.5 | 3.5 | 2.1 | 0.7 | **16** | **I** |
|  | FLZ-Cu | 84.3 | 85.3 | 83.7 | 76.0 | 51.3 | 28.3 | 11.9 | 6.3 | 4.9 | 3.7 | 2.8 | **8** |  |
|  | Cu(II) | 30.5 | 19.0 | 15.1 | 9.0 | 6.1 | 5.8 | 4.7 | 4.0 | 3.7 | 3.1 | 2.3 | **-** |  |
| *C. glabrata* 2231 | FLZ | 90.5 | 90.8 | 81.0 | 47.6 | 28.4 | 18.8 | 9.2 | 6.5 | 5.2 | 3.2 | 2.7 | **32** | **I** |
|  | FLZ-Cu | 84.2 | 83.9 | 83.9 | 75.7 | 47.3 | 24.7 | 11.5 | 5.4 | 3.7 | 3.2 | 3.2 | **16** |  |
|  | Cu(II) | 26.0 | 15.7 | 10.6 | 8.1 | 5.4 | 4.5 | 3.4 | 3.6 | 2.9 | 3.1 | 2.4 | **-** |  |
| *C. glabrata* 2232 | FLZ | 78.2 | 77.6 | 75.7 | 62.7 | 32.8 | 20.1 | 13.8 | 9.6 | 7.2 | 5.5 | 4.3 | **16** | **I** |
|  | FLZ-Cu | 86.5 | 81.9 | 75.1 | 66.1 | 53.8 | 32.6 | 17.2 | 8.7 | 5.4 | 4.2 | 3.8 | **8** |  |
|  | Cu(II) | 30.9 | 22.5 | 9.8 | 5.2 | 2.7 | 2.7 | 2.6 | 2.4 | 2.2 | 2.1 | 1.9 | **-** |  |
| *C. glabrata* 2233 | FLZ | 53.4 | 53.1 | 52.8 | 48.2 | 35.0 | 23.5 | 11.8 | 6.0 | 5.4 | 3.4 | 2.2 | **32** | **I** |
|  | FLZ-Cu | 76.9 | 68.0 | 59.4 | 51.2 | 34.3 | 20.9 | 8.4 | 3.8 | 2.6 | 1.8 | 1.2 | **16** |  |
|  | Cu(II) | 32.5 | 20.1 | 16.5 | 6.7 | 5.1 | 5.2 | 4.2 | 3.7 | 3.0 | 2.0 | 1.2 | **-** |  |
| *C. glabrata* 2235 | FLZ | 92.5 | 85.9 | 36.7 | 10.0 | 7.0 | 5.8 | 5.5 | 4.7 | 3.9 | 3.2 | 2.3 | **64** | **R** |
|  | FLZ-Cu | 91.6 | 88.3 | 43.4 | 15.0 | 5.4 | 3.9 | 3.2 | 3.3 | 3.0 | 3.6 | 1.2 | **64** |  |
|  | Cu(II) | 26.6 | 18.9 | 13.1 | 6.7 | 4.1 | 5.2 | 3.7 | 3.3 | 3.9 | 3.6 | 2.9 | **-** |  |
| *C. albicans* 2181 | FLZ | 87.6 | 87.0 | 86.5 | 85.3 | 59.3 | 32.4 | 16.1 | 9.0 | 6.6 | 4.5 | 2.6 | **8** | **R** |
|  | FLZ-Cu | 72.7 | 70.8 | 62.8 | 62.6 | 60.3 | 35.9 | 16.7 | 6.5 | 5.5 | 3.9 | 2.5 | **8** |  |
|  | Cu(II) | 35.5 | 20.4 | 9.4 | 6.5 | 5.0 | 4.5 | 3.9 | 2.8 | 2.6 | 2.6 | 1.2 | **-** |  |
| *C. albicans* 2200 | FLZ | 87.7 | 86.7 | 86.4 | 86.2 | 85.8 | 85.2 | 83.8 | 83.6 | 80.4 | 79.7 | 63.2 | **0.125** | **S** |
|  | FLZ-Cu | 88.6 | 86.7 | 86.4 | 86.2 | 85.9 | 85.4 | 83.9 | 83.0 | 81.3 | 77.0 | 63.7 | **0.125** |  |
|  | Cu(II) | 24.2 | 16.7 | 15.4 | 14.9 | 14.0 | 11.6 | 10.4 | 9.0 | 8.7 | 8.7 | 7.8 | **-** |  |
| *C. albicans* 2201 | FLZ | 91.4 | 89.7 | 88.9 | 88.6 | 88.9 | 89.1 | 88.3 | 89.0 | 84.4 | 79.4 | 44.2 | **0.25** | **S** |
|  | FLZ-Cu | 86.6 | 87.9 | 88.6 | 88.3 | 86.4 | 88.3 | 87.7 | 84.1 | 85.1 | 74.7 | 44.6 | **0.25** |  |
|  | Cu(II) | 16.4 | 12.5 | 8.1 | 8.8 | 5.9 | 3.7 | 3.1 | 3.1 | 4.6 | 3.5 | 5.4 | **-** |  |
| *C. albicans* 2203 | FLZ | 89.5 | 89.3 | 89.2 | 89.0 | 88.4 | 87.7 | 87.0 | 86.9 | 85.3 | 76.0 | 71.8 | **0.125** | **S** |
|  | FLZ-Cu | 89.2 | 88.3 | 87.9 | 87.7 | 85.1 | 81.5 | 81.0 | 80 | 79.5 | 58.5 | 13.8 | **0.25** |  |
|  | Cu(II) | 18.7 | 9.2 | 8.7 | 8.4 | 7.7 | 7.0 | 5.3 | 4.8 | 4.0 | 2.9 | 2.7 | **-** |  |
| *C. albicans* 2204 | FLZ | 83.4 | 80.7 | 80.4 | 74.2 | 51.1 | 22.2 | 12.6 | 10.3 | 8.1 | 5.2 | 3.7 | **8** | **R** |
|  | FLZ-Cu | 84.3 | 80.3 | 76.7 | 74.1 | 60.7 | 29.4 | 9.5 | 4.7 | 4.4 | 4.2 | 1.7 | **8** |  |
|  | Cu(II) | 30.7 | 22.4 | 10.8 | 4.9 | 1.3 | 1.6 | 1.9 | 0.7 | 0.7 | 0.6 | 0.2 | **-** |  |
| *C. albicans* 2208 | FLZ | 88.9 | 89.2 | 88.3 | 88.4 | 88.6 | 87.4 | 86.6 | 85.6 | 81.8 | 73.0 | 32.1 | **0.25** | **S** |
|  | FLZ-Cu | 87.6 | 87.1 | 87.3 | 86.6 | 86.6 | 85.6 | 85.5 | 84.7 | 81.4 | 31.2 | 19.5 | **0.5** |  |
|  | Cu(II) | 26.1 | 26.9 | 26.2 | 27.6 | 26.3 | 27.9 | 23.0 | 19.0 | 17.0 | 15.8 | 15.5 | **-** |  |
| *C. albicans* 2209 | FLZ | 79.2 | 77.0 | 76.9 | 75.0 | 73.4 | 73.8 | 71.4 | 71.5 | 65.6 | 40.8 | 6.5 | **0.5** | **S** |
|  | FLZ-Cu | 80 | 79.2 | 79.0 | 79.2 | 78.7 | 78.5 | 77.2 | 73.1 | 70.3 | 64.8 | 22.9 | **0.25** |  |
|  | Cu(II) | 24.8 | 19.0 | 19.3 | 17.4 | 14.2 | 12.0 | 10.5 | 9.3 | 7.7 | 6.2 | 6.7 | **-** |  |
| *C. albicans* 2210 | FLZ | 88.4 | 88.5 | 87.5 | 86.5 | 86.2 | 86.8 | 86.2 | 84.1 | 54.4 | 29.4 | 8.4 | **0.125** | **S** |
|  | FLZ-Cu | 88.2 | 87.8 | 88.0 | 86.8 | 87.3 | 87.1 | 86.0 | 84.4 | 81.6 | 63.7 | 34.7 | **0.25** |  |
|  | Cu(II) | 28.4 | 18.4 | 12.0 | 7.9 | 5.7 | 5.1 | 5.0 | 3.6 | 3.2 | 3.9 | 2.6 | **-** |  |
| *C. albicans* 2211 | FLZ | 90.6 | 90.2 | 90.5 | 89.8 | 89.4 | 89.1 | 88.5 | 86.7 | 85.8 | 27.1 | 16.4 | **0.5** | **S** |
|  | FLZ-Cu | 90.9 | 89.8 | 89.6 | 89.4 | 89.1 | 89.3 | 89.1 | 89.1 | 87.4 | 73.6 | 15.8 | **0.25** |  |
|  | Cu(II) | 28.3 | 23.6 | 21.6 | 18.7 | 12.7 | 12.8 | 10.2 | 11.8 | 11.2 | 9.5 | 8.9 | **-** |  |
| *C. albicans* 2212 | FLZ | 85.2 | 85.4 | 84.9 | 84.1 | 85.2 | 85.6 | 83.7 | 83.3 | 78.1 | 39.9 | 9.7 | **0.5** | **S** |
|  | FLZ-Cu | 89.0 | 87.2 | 88.2 | 89.2 | 88.2 | 88.9 | 88.5 | 87.8 | 86.6 | 77.2 | 11.3 | **0.25** |  |
|  | Cu(II) | 28.9 | 28.2 | 25.8 | 25.4 | 20.7 | 18.7 | 18.7 | 17.5 | 15.6 | 12.6 | 8.0 | **-** |  |
| *C. albicans* 2213 | FLZ | 73.7 | 73.5 | 73.4 | 73.3 | 73.5 | 72.8 | 72.9 | 72.5 | 70.3 | 60.2 | 48.6 | **0.25** | **S** |
|  | FLZ-Cu | 79.1 | 73.2 | 73.2 | 71.1 | 71.3 | 71.8 | 70.0 | 69.4 | 69.9 | 65.3 | 57.8 | **0.125** |  |
|  | Cu(II) | 13.0 | 7.9 | 6.2 | 6.2 | 5.0 | 4.9 | 3.5 | 3.0 | 2.9 | 2.7 | 1.5 | **-** |  |
| *C. albicans* 2214 | FLZ | 82.0 | 81.0 | 80.4 | 80.5 | 79.9 | 79.5 | 79.3 | 78.6 | 78.3 | 73.5 | 54.3 | **0.125** | **S** |
|  | FLZ-Cu | 87.2 | 87.2 | 84.9 | 82.4 | 81.5 | 80.2 | 80.2 | 79.9 | 79.9 | 71.5 | 28.3 | **0.25** |  |
|  | Cu(II) | 16.6 | 12.2 | 10.5 | 10.8 | 10.1 | 10.1 | 10.3 | 7.7 | 7.6 | 6.8 | 5.4 | **-** |  |
| *C. albicans* 2215 | FLZ | 84.6 | 84.2 | 84.2 | 83.8 | 83.7 | 83.0 | 82.7 | 81.4 | 78.0 | 67.8 | 37.4 | **0.25** | **S** |
|  | FLZ-Cu | 90.5 | 89.5 | 81.8 | 81.2 | 81.4 | 80.5 | 79.3 | 78.1 | 77.4 | 73.9 | 54.0 | **0.125** |  |
|  | Cu(II) | 28.8 | 19.4 | 15.3 | 9.4 | 5.7 | 6.6 | 6.0 | 5.3 | 5.2 | 4.1 | 3.6 | **-** |  |
| *C. albicans* 2216 | FLZ | 92.3 | 92.2 | 92.0 | 92.0 | 92.0 | 92.1 | 91.9 | 91.7 | 91.2 | 87.8 | 50.6 | **0.125** | **S** |
|  | FLZ-Cu | 91.8 | 91.9 | 91.6 | 91.4 | 91.5 | 91.6 | 91.4 | 91.3 | 91.0 | 89.3 | 57.0 | **0.125** |  |
|  | Cu(II) | 71.6 | 63.8 | 50.3 | 31.6 | 17.6 | 13.5 | 13.4 | 12.0 | 11.8 | 11.2 | 7.0 | **-** |  |
| *C. albicans* 2218 | FLZ | 78.1 | 72.1 | 64.9 | 69.6 | 60.9 | 43.5 | 28.2 | 15.5 | 12.0 | 10.4 | 7.4 | **8** | **R** |
|  | FLZ-Cu | 89.0 | 60.2 | 51.0 | 46.7 | 44.0 | 36.3 | 24.5 | 12.5 | 9.7 | 7.9 | 5.4 | **32** |  |
|  | Cu(II) | 31.2 | 20.6 | 11.4 | 6.3 | 3.5 | 3.9 | 3.2 | 3.2 | 3.5 | 3.6 | 3.1 | **-** |  |
| *C. albicans* P6 | FLZ | 88.0 | 86.1 | 86.1 | 83.6 | 75.6 | 61.4 | 56.3 | 51.8 | 48.4 | 45.4 | 38.3 | **1** | **S** |
|  | FLZ-Cu | 85.3 | 84.7 | 84.7 | 82.5 | 76.9 | 67.1 | 54.3 | 53.2 | 47.2 | 44.0 | 41.0 | **1** |  |
|  | Cu(II) | 17.7 | 12.5 | 11.3 | 11.4 | 11.4 | 11.1 | 9.5 | 9.2 | 9.3 | 8.5 | 7.3 | **-** |  |

* Concentration for FLZ; ** Concentration for FLZ-Cu; *** Concentration for Cu(II); the highlighted orange background concern discussed strains in main text
